# 

**Published:** 1866-07

**Authors:** 


### Books and Pamphlets Received.

Medical Electricity—Embracing Electro-Physiology and Electricity as a Therapeutic, with special reference to practical medicine, showing the most approved apparatus, method and rules for the medical uses of Electricity in the treatment of nervous diseases. By Alfred C. Garratt, M. D., Fellow of the Massachusetts Medical Society, member of the American Medical Association. Third edition, revised and illustrated. Philadelphia: J. B. Lippincott & Co., 1866.

Medical Diagnosis with special reference to Practical Medicine. A guide to the knowledge and discrimination of diseases. By J. M. Da Costa, Lecturer on Clinical Medicine, and Physician to the Pennsylvania Hospital; Fellow of the College of Physicians of Philadelphia; Corresponding Member of the New York Pathological Society, etc., etc., illustrated with engravings on wood. Second edition, revised. Philadelphia: J. B. Lippincott & Co., 1866.

